# Clinical Use of Lecanemab at an Academic Medical Center

**DOI:** 10.1002/alz.095181

**Published:** 2025-01-09

**Authors:** Lawrence S. Honig, Jee Min Kim, Wendy P. Gonzalez, Vanessa DiMuro, Radhika Jagannathan, Karen Marder, James M. Noble, Karen L. Bell, Richard Mayeux

**Affiliations:** ^1^ Columbia University Irving Medical Center, New York, NY USA

## Abstract

For Alzheimer’s disease (AD), there are currently two FDA‐approved agents developed as disease‐modifying treatments. Approval of lecanemab (Leqembi®) in 2023, via accelerated approval mechanism, followed by traditional approval accompanied by medical coverage decision by the Center for Medicare Services, has resulted in increasing use of this anti‐amyloid monoclonal antibody, demonstrated in 18‐month long clinical trials to slow Alzheimer’s disease. Despite a broad package insert (with only contraindication product severe hypersensitivity), nationwide prescriptions have been reportedly affected by concerns regarding drug eligibility, monitoring, and whether real‐world experience would mirror clinical trial data. In our practice, prescription of the medication has been contingent upon: (a) diagnosis of AD, (b) early stage of AD, (c) amyloid confirmation by CSF or PET, (d) acceptable ARIA‐H at baseline MRI, and (e) non‐mandatory apolipoprotein‐E genotyping. Here we present data on the first 85 patients prescribed lecanemab at our center. Patient age is 73±8 yr (51‐88) and sex 51% women, with 82% accepting apolipoprotein‐E4 testing (42% non‐carriers, 44% heterozygotes, and 14% homozygotes). Barriers associated with our above eligibility criteria (a) through (e) have been minimal. Similarly, barriers have generally not been large for MRI monitoring and infusion center availability; but during this first year of drug availability there have been challenges in coverage by insurers/payors other than standard original Medicare. Patients/families have had strong interest in this drug, with apparent good understanding and acceptance of risks of ARIA‐E and ARIA‐H. Patients deemed not eligible by their treating practitioners have been declined for the following reasons in descending order of frequency: disease too severe, non‐Alzheimer’s dementia (no amyloid confirmation), logistical (travel/time) considerations, disease too mild (presymptomatic), and excessive ARIA‐H on baseline MRI. Overall, with up to 21 infusions per patient, adverse event rates have been similar to those in clinical trials with 7% ARIA–E (all asymptomatic), and low rates of infusion reactions. Suspension or discontinuation of therapy has occurred in 9% (7% due to ARIA, 1% due to persistent infusion reactions, 2% due to disinterest). Overall findings at our center suggest that experience in clinical use may not be dissimilar to that in the clinical trials.

